# The mediating role of mentalizing capacity between parents and peer attachment and adolescent borderline personality disorder

**DOI:** 10.1186/s40479-017-0074-4

**Published:** 2017-11-24

**Authors:** Emma Beck, Carla Sharp, Stig Poulsen, Sune Bo, Jesper Pedersen, Erik Simonsen

**Affiliations:** 1 Psychiatric Research Unit, Region Zealand, Fælledvej 6, 4200 Slagelse, Denmark; 2 Child and Adolescent Psychiatric Department, Region Zealand, Smedegade 16, 4000 Roskilde, DK Denmark; 3 Department of Psychology, University of Copenhagen, 2A Øster Farimagsgade, -1353 Copenhagen K, DK Denmark; 4 Department of Psychology, University of Houston, Houston, TX 77204 USA; 5 Institute of Clinical Medicine, Faculty of Health and Medical Sciences, University of Copenhagen, Copenhagen, Denmark

**Keywords:** Attachment to peers, Adolescent borderline personality disorder, Reflective function, Mentalization, Personality pathology

## Abstract

**Background:**

Insecure attachment is a precursor and correlate of borderline personality disorder. According to the mentalization-based theory of borderline personality disorder, the presence of insecure attachment derails the development of the capacity to mentalize, potentially resulting in borderline pathology. While one prior study found support for this notion in adolescents, it neglected a focus on peer attachment. Separation from primary caregivers and formation of stronger bonds to peers are key developmental achievements during adolescence and peer attachment warrants attention as a separate concept.

**Findings:**

In a cross-sectional study, female outpatients (M_age_ 15.78=, SD = 1.04) who fulfilled DSM-5 criteria for BPD (*N* = 106) or met at least 4 BPD criteria (*N* = 4) completed self-reports on attachment to parents and peers, mentalizing capacity (reflective function) and borderline personality features. Our findings suggest that in a simple mediational model, mentalizing capacity mediated the relation between attachment to peers and borderline features. In the case of attachment to parents, the mediational model was not significant.

**Conclusions:**

The current study is the first to evaluate this mediational model with parent and peer attachment as separate concepts and the first to do so in a sample of adolescents who meet full or sub-threshold criteria for borderline personality disorder. Findings incrementally support that mentalizing capacity and attachment insecurity, also in relation to peers, are important concepts in theoretical approaches to the development of borderline personality disorder in adolescence. Clinical implications are discussed.

## Background

Borderline personality disorder (BPD) typically develops in adolescence [1], and has profound effects on developmental outcomes [2]. Identification of early correlates and precursors is important for the development of early intervention programs. Insecure attachment has been identified empirically as one such correlate of BPD in cross-sectional, retrospective, and prospective studies [3–5], with the insecure, preoccupied and unresolved attachment status being predominant among adults with BPD [3, 6]. In the mentalization-based theory of BPD, Fonagy and colleagues [7] proposed that a central feature of BPD is a profound impairment in the capacity to mentalize. Mentalization has been defined as a developmentally acquired capacity to understand and interpret - implicitly and explicitly - one’s own and others’ behavior as an expression of mental states such as feelings, thoughts, fantasies, beliefs, and desires [7]. Empirical investigations suggest that BPD is associated with impairments in specific aspects of mentalizing or reflective function [8–11], with these two terms often being used interchangeably in the literature. The theory postulates that attachment security is the developmental context through which mentalization develops: A reflective caregiver stimulates attachment bonding and promotes secure attachment, which in turn fosters reflective function and optimal socio-emotional function in the child [12]. In BPD, it is thought that the presence of insecure attachment derails the development of mentalizing, potentially resulting in borderline pathology.

Very few studies have investigated the links between attachment, mentalizing and borderline features in adolescents [8, 13, 14]. To our knowledge, only Sharp and colleagues [15] directly investigated if mentalizing mediated the relation between attachment insecurity and borderline features and found support for this hypothesis. While important to establish proof of principle, their study neglected a focus on peer attachment. A key developmental achievement during adolescence is separation from primary caregivers and formation of stronger bonds to peers. As yet, it remains unclear how attachment to peers interrelate with mentalizing capacity in adolescent BPD. The present study builds incrementally on prior work by examining the mediating role of mentalizing in the relation between attachment to peers and parents and borderline features in a sample of adolescent BPD outpatients. More specifically, based on the literature we hypothesized that mentalizing would mediate the relationship between attachment to parents and to peers, respectively, and borderline features.

## Methods

### Participants

Data were collected at baseline in a randomized clinical trial on MBT for adolescent BPD, the M-GAB trial [16]. One hundred and eleven outpatients were recruited from four child and adolescent psychiatric outpatient clinics, with only one male who was excluded from the subsequent analyses. One hundred and ten female participants (M_age_ 15.78=, SD = 1.04) fulfilled DSM-5 criteria for BPD (*N* = 106) or met at least four BPD criteria (*N* = 4) using the semi-structured Childhood Interview for DSM-IV Borderline Personality Disorder (CI-BPD). For further inclusion and exclusion criteria, see Beck et al. 2016 [16].

### Measures

*The Borderline Personality Features Scale for Children (BPFS-C)* [17] is a 24-item measure rated on a 5-point Likert scale. Crick et al. [17] established evidence for the construct validity and demonstrated high internal consistency. In this sample, internal consistency was good with a Cronbach’s alpha of .85.

*The Inventory of Parent and Peer Attachment-Revised (IPPA-R)* [18]. The IPPA-R is a 53-item measure of attachment in adolescence. It comprises two scales that measure attachment to parents and peers, respectively, and has shown good validity, reliability and psychometric properties [18]. In this sample, internal consistency was excellent with Cronbach’s alpha of .94 for IPPA-Parents and .93 for IPPA-Peers.

*Reflective Function Questionnaire for Youth* (RFQ-Y) is a 46-item self-report questionnaire measuring the general capacity to mentalize. It has shown good psychometric properties, including construct validity [19]. In the present sample, internal consistency was acceptable with Cronbach’s alpha of .75.

The *CI-BPD* was used to determine whether participants met inclusion criteria for the study (≥ 4 or more criteria of BPD) [20].

*The Youth Self-Report (YSR)* [21] is a 112-item self-report measure of general psychopathology rated on a 3-point Likert scale for use with adolescents between 11 and 18 years. Total problem T-scores were used. In the present sample, internal consistency was excellent with Cronbach’s alpha of .93.

## Results

### Descriptive results and bivariate relations

Descriptive statistics are presented in Table 1. As expected for a sample of adolescents who meet ≥4 criteria of BPD, participants showed clinically significant levels on the BPFS-C (i.e. > 65; [22]).

**Table 1:** Descriptive information for each study variable

Measure	Mean	SD
Borderline Personality Features (BPFS-C)	79.79	12.15
Attachment to Parents (IPPA Parent)	52.26	11.81
Attachment to Peers (IPPA Peer)	43.24	9.60
Reflective Function (RFQ-Y)	6.18	.60
General Psychopathology (YSR)	73.85	10.90
Age	15.78	1.04

Pearson’s correlations between key study variables are presented in Table 2. These analyses revealed that more severe borderline features were significantly associated with lower mentalizing capacity, lower levels of attachment security to both parent and peers and increased level of general psychopathology. Age was not significantly related to any other variable and was therefore not included as a covariate in subsequent analyses.

**Table 2:** Pearson correlations between key study variables

	BPFSC	IPPA Parent	IPPA Peer	RFQ-Y	YSR
IPPA Parent	.329^a^				
IPPA Peer	.338^a^	.387^a^			
RFQ-Y	-.547^a^	−.181	-.269^b^		
YSR	.664^a^	.351^a^	.254^b^	-.362^a^	
Age	.001	.026	−.030	−.035	−.035

^a^Correlation is significant at the 0.01 level (2-tailed). ^b^Correlation is significant at the 0.05 level (2-tailed)

### Mediational analyses

First, we conducted Preacher and Hayes’ test of the indirect effect [23] to determine if mentalizing (RFQ-Y) mediated the relation between attachment to peers (IPPA-R Peers) as the independent variable and borderline features (BPFS-C) as the dependent variable. The result indicated the presence of a significant mediational effect, with the mean of the indirect effect across all bootstrap samples estimated at 0.17 and a resulting confidence interval that did not include 0 (*CI* = 0.04 to 0.35; [23]). Unstandardized path coefficients for this mediational model are presented in Table 3. Multicollinearity was not a problem with a tolerance of 0.93 [24, 25]. In order to ensure specificity of a relation to BPD in particular, the mediational analysis was repeated with general psychopathology (externalizing and internalizing combined) as covariate. The result of this model also indicated the presence of a significant mediational effect, with the mean of the indirect effect across all bootstrap samples estimated at 0.07 and a resulting confidence interval that did not include 0 (*CI* = 0.0016 to 0.2310; [23]). In this model, multicollinearity was not a problem either with tolerance greater than 0.83 in all cases.

**Table 3:** Mediational model of the effect of attachment to Peers on borderline features through reflective function

Path	Coefficient	*SE*	*p*
Model 1			
A. IV IPPA-Peers to Mediator RFQ-Y	−.02	.007	.02
B. Mediator RFQ-Y to DV Borderline Features	−10.36	1.86	.000
C. Total Effect: IPPA-Peers to Borderline Features	.39	.13	.004
C′. Direct Effect: IPPA-Peers to Borderline Features	.22	.12	.06

*IV* Independent Variable. *DV* Dependent Variable. Coefficient Unstandardized path coefficient. *SE* Standard error. *IPPA-Peers* The Inventory of Parent and Peer Attachment - Revised (IPPA-R) for children. *RFQ-Y* the Reflective Function Questionnaire for Youths (RFQY)

When the analysis was repeated with attachment to parents as the independent variable, the model was no longer significant with the mean of the indirect effect across all bootstrap samples estimated at 0.1 and a resulting confidence interval that did include 0 (*CI* = **−** 0.008 to 0.24; [23]). Since the cross-sectional nature of the data precludes strong conclusions about causality, we tested directionality by examining a reversed model in which the indirect effects of mentalizing capacity on borderline features were explored using attachment insecurity to peers as the mediator. This model did not confirm the mediating effect of attachment insecurity to peers on the relation between mentalizing and borderline features, with the mean of the indirect effect across all bootstrap samples estimated at −0.94 and a confidence interval that included 0 (*CI* = − 3.57 to 0.19). In all the conducted mediational analyses, 1000 bootstrap samples were drawn.

### Conclusions

The aim of this study was to further evaluate the hypothesis that attachment security relates to borderline features via mentalizing capacity. While one prior study found support for this notion, this is the first study to evaluate these links for peer attachment and the first to do so in a sample of adolescents with borderline pathology. Findings support that attachment to peers is significantly related to borderline features through mentalizing as a mediator.

In adolescence, peer relationships gradually take on qualities of adult reciprocal attachment relationships and move up the attachment hierarchy [26, 27] possibly serving as an important developmental link between the parent-child and later adult romantic attachment relationships [28]. Studies suggest that in general, adolescent attachment security is linked to mental health [26], and that attachment to peers may play a particular important role [27], also in relation to borderline pathology [14]. A clinical implication of this study for the treatment of adolescent BPD may be the possible benefits of targeting mentalizing specifically in the context of reciprocal peer relationships, be it in the here-and-now of group psychotherapy [16] or in relation to peer relationships external to the treatment context.

The relatively high degree of homogeneity of the sample and resulting low variance in the target variables may explain why mentalizing was not a significant mediator between attachment to parents and borderline features. Future research would benefit from including participants with low BPD features to increase variance. Concerning the difference found between parents and peers, the direct relation between RFQ-Y and IPPA-Parents is significant with an effect size only a little lower than the one found for IPPA-Peers (−.18 vs. -.27). One possible explanation for this weaker association is that a large proportion of the RFQ-Y items specifically describe the relationship between the self and “people”, i.e. “I get confused when people talk about their feelings.” “People” as a concept may be closer in meaning to the relatively non-specific concept of “friends” used in IPPA-Peer compared to the concept of “parents” in IPPA-Parents, and thereby the aspects of mentalizing measured by the RFQ-Y may be stronger associated with peer attachment contexts than with caregiver-attachment contexts.

Important limitations of this study include: 1) All measures are based on self-report and therefore subject to shared method variance, and 2) The design is cross-sectional and correlational in nature and therefore conclusions about causation are unwarranted. Despite these limitations, findings incrementally support that mentalizing capacity and attachment insecurity, also in relation to peers, are important concepts in theoretical approaches to the development of BPD in adolescence.

## Abbreviations

BPD: Borderline personality disorder
